# Hospital Meetings

**Published:** 1901-02-02

**Authors:** 


### The Institutional Workshop.

### HOSPITAL MEETINGS.

### ITALIAN HOSPITAL.

THE annual general meeting of the Italian Hospital was held for the first time in the new building, Queen Square, on Saturday last, January 26th. The chair was taken by Cav. Dr. M. Castaneda. The report presented stated that the past year had been a marked period in the history of the hospital, and the committee expressed satisfaction that the wishes and aspirations with which their last report closed had in an encouraging manner been realised, the hospital in its new home and extended sphere having already proved its need and value. The new buildings were opened on March 14th last by the late Baron de Renzis, the Italian Ambassador, who presented to Mrs. Ortelli, the widow of the Founder, a commemorative medallion of gold set with diamonds, from King Humbert. Since that ceremony both His Majesty and His Excellency had passed away, the hospital thus losing its Patron and its President within three months. The temporary hospital in Orange Street was closed on February 10th, and the new buildings opened to out-patients on April 2nd, and to in-patients on May 1st, since which date 215 in- and 4,673 out-patients had been relieved. Of the total of 4,888, 2,224 were British subjects. The hospital has accommodation for 50 in-patients, but the committee only felt justified in opening a portion of the beds until the funds should prove sufficient for the full work without incurring debt. After much consideration the committee felt that the annual festival ball would ill accord with the sorrows and anxieties caused by the South African war, and though the hospital depended largely on it for the necessary help to carry on the work, it was resolved, as a mark of sympathy with England, to abandon the ball. The annual subscriptions amounted to £371, as compared with £269 in the previous year. The donations also showed an increase of £376. The total ordinary receipts were £1,829, the total ordinary expenditure £2,209, and there was thus a deficit of close on £400. Attention was drawn to the fact that the Swiss Government had decided to make an annual grant of 200fr. in recognition of the hospital's work among Swiss residents in England. Lord Rosebery also gave a donation of £50. With a view to meeting the increased requirements of the hospital, the committee decided to appoint an assistant hon. physician, and Dr. Vincent Dickinson was selected. Dr. J. P. Henry was appointed ophthalmic surgeon, and Dr. Bulger anæsthetist. Consequent upon these additions to the staff, Cav. Dr. M. Castaneda was elected a consulting physician, Cav. Dr. F. Naumann succeeding him as senior physician. On the resignation of Dr. Treston, Dr. E. D. Pereira was appointed resident medical officer. At the first meeting of the committee in the new buildings, Mr. W. E. Cutler, F.R.I.B.A., the architect, was unanimously elected consulting architect to the hospital.

Before submitting the report, the chairman proposed a vote of condolence on the death of the Queen "beloved by the world, and whose memory will be revered in many lands." This was agreed to and the report adopted. Votes of thanks concluded the proceedings.

